# Characterization of an oligometastatic state in patients with metastatic pancreatic adenocarcinoma undergoing systemic chemotherapy

**DOI:** 10.1002/cam4.6582

**Published:** 2023-12-22

**Authors:** Saryleine de Ortiz de Choudens, Alexis Visotcky, Anjishnu Banerjee, Mohammed Aldakkak, Susan Tsai, Douglas B. Evans, Kathleen K. Christians, Callisia N. Clarke, Ben George, Aditya Shreenivas, Mandana Kamgar, Sakti Chakrabarti, Kulwinder S. Dua, Abdul Haq Khan, Srivats Madhavan, Beth A. Erickson, William A. Hall

**Affiliations:** ^1^ Department of Radiation Oncology Medical College of Wisconsin Milwaukee Wisconsin USA; ^2^ Division of Biostatistics Medical College of Wisconsin Milwaukee Wisconsin USA; ^3^ Department of Surgery Medical College of Wisconsin Milwaukee Wisconsin USA; ^4^ LaBahn Pancreatic Cancer Program Milwaukee Wisconsin USA; ^5^ Division of Medical Oncology Medical College of Wisconsin Milwaukee Wisconsin USA; ^6^ Division of Gastroenterology Medical College of Wisconsin Milwaukee Wisconsin USA

**Keywords:** neoplasm metastasis, oligometastasis, pancreatic neoplasms

## Abstract

**Purpose/Objectives:**

Most patients with pancreatic adenocarcinoma (PDAC) will present with distant metastatic disease at diagnosis. We sought to identify clinical characteristics associated with prolonged overall survival (OS) in patients presenting with metastatic PDAC.

**Materials/Methods:**

Patients presenting with metastatic PDAC that received treatment at our institution with FOLFIRINOX or gemcitabine‐based chemotherapies between August 1, 2011 and September 1, 2017 were included in the study. Metastatic disease burden was comprehensively characterized radiologically via individual diagnostic imaging segmentation. Landmark analysis was performed at 18 months, and survival curves were estimated using the Kaplan–Meier method and compared between groups via the log‐rank test. ECOG and Charlson Comorbidity Index (CCI) were calculated for all patients.

**Results:**

121 patients were included with a median age of 62 years (37–86), 40% were female, 25% had ECOG 0 at presentation. Of the 121 patients included, 33% (*n* = 41) were alive at 12 months and 25% (*n* = 31) were alive at 18 months. Landmark analysis demonstrated a significant difference between patients surviving <18 months and ≥18 months regarding the presence of lung only metastases (36% vs. 16%, *p* = 0.04), number of organs with metastases (≥2 vs. 1, *p* = 0.04), and disease volume (mean of 19.1 cc vs. 1.4 cc, *p* = 0.04). At Year 1, predictors for improved OS included ECOG status at diagnosis (ECOG 0 vs. ECOG 1, *p* = 0.04), metastatic disease volume at diagnosis (≤0.1 cc vs. >60 cc, *p* = 0.004), metastasis only in the liver (*p* = 0.04), and normalization of CA 19‐9 (*p* < 0.001). At Year 2, the only predictor of improved OS was normalization of the CA 19‐9 (*p* = 0.03). In those patients that normalized their CA 19‐9, median overall survival was 16 months.

**Conclusions:**

In this exploratory analysis normalization of CA‐19‐9 or volumetric metastatic disease burden less than 0.2 cc demonstrated a remarkable OS, similar to that of patients with non‐metastatic disease. These metrics are useful for counseling patients and identifying cohorts that may be optimal for trials exploring metastatic and/or local tumor‐directed interventions.

## Graphical abstract

In this study, we sought to identify clinical characteristics associated with prolonged overall survival (OS) in patients presenting with metastatic pancreatic ductal adenocarcinoma. In this exploratory analysis, normalization of CA‐19‐9 or volumetric metastatic disease burden less than 0.2 cc demonstrated a remarkable OS, similar to that of patients with non‐metastatic disease. At Year 1, predictors for improved OS included ECOG status at diagnosis, metastatic disease volume at diagnosis, metastasis only in the liver, and normalization of CA 19‐9.

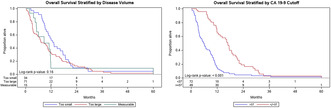

## 1 INTRODUCTION

Pancreatic adenocarcinoma (PDAC) is a highly aggressive malignancy and will become the second leading cause of cancer‐related death in the United States by 2030.^1^, ^2^ Many patients with PDAC present with clear radiological evidence of metastatic disease. Even in those PDAC patients without overt radiological evidence of metastatic disease, patients are often believed to harbor micro‐metastatic disease at the time of diagnosis.^3^, ^4^, ^5^, ^6^ For this reason, neoadjuvant systemic chemotherapy is an increasingly common strategy for all stages of PDAC.^7^ This approach serves to eliminate micro‐metastatic disease and identify those patients without evidence of metastatic disease, who may benefit from local interventions on their primary tumor, such as radiation therapy or surgery. There are increasing clinical outcomes publications regarding the efficacy and safety of this approach in patients without evidence of metastatic disease.^8^, ^9^

Patients presenting with overt radiological evidence of metastatic PDAC are often relegated to non‐curative therapy, often with chemotherapy alone. This results in a dismal prognosis, with median overall survival ranging from 6 to 11 months, despite modern chemotherapy.^10^ There are clinical anecdotes of long‐term survivors with metastatic PDAC, but their characteristics remain elusive. It remains unknown as to the precise disease characteristics in patients with longer durations of OS who may warrant clinical trial enrollment examining more aggressive management of the primary tumor or metastatic disease.^11^

Patients presenting with overt radiological evidence of distant metastatic disease are known to be a heterogeneous cohort. It remains poorly understood if some patients presenting with radiological apparent metastatic disease should be considered for more aggressive treatment approaches, such as focused radiation therapy or even surgical resection. In other solid tumors, numerous prospective trials are moving toward a more nuanced understanding of metastatic disease as existing along a spectrum.^12^, ^13^ This spectrum includes some patients with a unique biological entity that has historically been referred to as “oligometastatic disease” first proposed by Hellman and Weichselbaum in 1995.^14^ Several clinical trials have shown improvement in OS with local treatments of the primary cancer, along with other sites of metastatic disease, in cohorts of patients with limited volume oligometastatic disease.^15^, ^16^, ^17^ These trials have very poorly represented patients with metastatic PDAC. Considering the combination of chemotherapy and local therapies, a new approach to metastatic disease in PDAC has become the focus of current research.^4^, ^6^ Retrospective data suggest that resection of the primary tumor and solitary liver metastases can potentially improve overall survival.^18^, ^19^ These encouraging results suggest a hypothesis that a select subset of metastatic PDAC patients could stand to benefit from definitive interventions beyond chemotherapy.^6^ We sought to identify characteristics associated with prolonged OS in patients presenting with de novo metastatic disease. The primary objective of this research is to identify cohorts of patients that may benefit from more aggressive local therapies.

## 2 METHODS

Clinical outcomes for patients presenting with metastatic PDAC at Froedtert and the Medical College of Wisconsin between 8/1/2011 and 9/1/2017 were collected retrospectively. TriNetX, a global health research network, was used to provide access to identify eligible patients and subsequently access electronic medical records. Patients were identified using ICD 10‐based queries (C25). Patients had to be diagnosed with de novo metastatic PDAC and received treatment with 5‐fluorouracil or gemcitabine‐based chemotherapies. Patients with localized disease that received definitive treatments at diagnosis and progressed to metastatic disease were excluded.

Study data was collected and managed using REDCap electronic data capture tools hosted at the Medical College of Wisconsin (MCW). This process was approved by the MCW institutional review board (PRO00037616).

### 2.1 Patient characteristics and inclusion criteria

All patients had histological confirmation of PDAC obtained via biopsy of metastases or the primary tumor. We retrospectively assessed clinical data, including sex, age at diagnosis, chemotherapy regimens received, tumor markers at diagnosis, and after first‐line chemotherapy (CA 19‐9 and CEA), laboratory parameters (LDH, bilirubin, CRP), Charlson Comorbidity Index (CCI), number of metastases, sites of metastases, metastatic disease burden, and time of death or last follow‐up date available. As cholestasis can influence the CA 19‐9 value, we included baseline CA 19‐9 when a patient had normalized bilirubin (<1.2 mg/dL) after stenting. Patients were required to have primary imaging data available in the picture archive and communication system (PACS) at the time of their original presentation of metastatic disease.

### 2.2 Characterization of metastatic disease burden

Metastatic disease burden was comprehensively characterized radiologically via imaging segmentation. This involved individual PACS queries for each patient of their original diagnostic imaging data. The location, number, and volume of metastatic disease (when able) were calculated using commercially available image annotation software (MIM Inc). ECOG performance status was obtained via chart review and CCI was calculated for all patients using publicly available online tools. The time of PDAC diagnosis was defined as the date of pathological confirmation of disease. The diagnosis of distant metastasis had to have been made at the time of initial diagnosis before the beginning of chemotherapy treatment. Follow‐up information was obtained from the institution's medical record system.

### 2.3 Statistical analysis

Overall survival was estimated using the Kaplan–Meier method. For categorical variables of interest, survival curves were compared using the log rank test. Estimates at 12 and 24 months are provided from these models. Descriptive statistics, including relative frequencies for demographic and clinical characteristics of patients were computed. Analyses were performed using SAS 9.4 (SAS Institute). Landmark analysis was performed at 18 months to identify characteristics associated with prolonged OS. This timeline was selected as it corresponds to two standard deviations above median OS. Characteristics evaluated included age, gender, CCI, ECOG performance status, tumor location, presence of regional lymphadenopathy, number, and location of metastatic lesions, volume of metastatic disease, mean value of CA 19‐9 and CEA, normalization of CA 19‐9, use of chemotherapy and use of local therapies.

## 3 RESULTS

### 3.1 Patient characteristics

121 patients were eligible for inclusion in this evaluation; 72 (60%) patients were male. The median age was 62 (37–86). Twenty‐nine patients (25%) had ECOG 0, and 60 patients (51%) had ECOG 1. 47 (39%) patients had a CCI of 0–2. Fifty‐three patients (44%) had a primary in the pancreatic head, 37 (31%) had a body lesion, and 30 (25%) had a lesion in the tail. Thirty‐seven (31%) patients had no evidence of lymphadenopathy in diagnostic images, while 84 (69%) patients had evidence of lymphadenopathy. Ninety patients (74%) had >10 metastatic lesions, while 30 patients (25%) had ≤10 metastatic lesions. Of the patients with ≤10 metastatic lesions, the median number of metastatic lesions was 3 (1.0–8.0). 37 patients (31%) had lung metastasis at diagnosis, while 90 patients (75%) had evidence of liver metastasis. The median number of organs with metastases was 1 (1.0–6.0). Disease volume was categorized as too small (<0.2 cc) in 34 patients, too large (>60.7 cc) in 71 patients, and measurable in 15 patients. For the patients with measurable disease, the median volume of disease was 9.6 cc. The CA 19‐9 mean was 9754, with a median of 775.4. The mean value of CEA was 31.9 with a median of 10. A complete list of patient's characteristics is provided in Table 1.

**TABLE 1:** Patient characteristics.

Variables	Total *N* = 121 (col %)
Gender	
Female	49 (40.5)
Male	72 (59.5)
Missing	0
Age	
*N*	121
Median (min–max)	62.0 (37.0–86.0)
Mean ± SD	62.2 ± 10.5
ECOG at diagnosis	
0 Fully active	29 (24.8)
1 Light activities	60 (51.3)
2 Limited but >50% OOB	25 (21.4)
3 Limited and <50% OOB	3 (2.6)
KPS at diagnosis	
100	1 (0.9)
90	34 (29.1)
80	55 (47.0)
70	22 (18.8)
60	5 (4.3)
Charlson Comorbidity Index	
0–2	47 (39.2)
3–4	46 (38.3)
5–6	21 (17.5)
7+	6 (5.0)
Tumor location	
Head	53 (44.2)
Body	37 (30.8)
Tail	30 (25.0)
Positive lymph nodes	
No	37 (30.6)
Yes	84 (69.4)
Fewer than 10 metastatic lesions	
No	90 (75.0)
Yes	30 (25.0)
Number of metastatic lesions (If fewer than 10)	
*N*	28
Median (min–max)	3.0 (1.0–8.0)
Mean ± SD	3.3 ± 2.0
Lung metastases	
No	84 (69.4)
Yes	37 (30.6)
Liver metastases	
No	31 (25.6)
Yes	90 (74.4)
Peritoneum metastases	
No	80 (66.1)
Yes	41 (33.9)
Disease volume	
<0.2 cc	34 (28.3)
>60.7 cc	71 (59.2)
CA19‐9	
Median (min–max)	775.4 (0.6–473,500.0)
Mean ± SD	9754.1 ± 47,146.0
Chemotherapy regimen	
FOLFIRINOX	48 (39.7)
FOLFOX	15 (12.4)
Xeloda (Capecitabine or 5FU)	1 (0.8)
Gemcitabine	10 (8.3)
Gem/nab‐paclitaxel	46 (38.0)
Gem/oxaliplatin	1 (0.8)

### 3.2 Chemotherapy regimens

Forty‐eight patients (40%) received first‐line treatment with FOLFIRINOX and 46 patients (38%) received first‐line treatment with gemcitabine + nab‐paclitaxel. Additional regimens used included FOLFOX, single‐agent 5FU, single‐agent gemcitabine, and gemcitabine‐oxaliplatin. The mean number of cycles was 7.3 ± 10.1. The median number of cycles was 4.0 (1.0–80.0). Forty‐seven patients (46.1%) had both primary tumor and metastatic responses.

### 3.3 Local therapies

Three patients had surgical resection of metastatic lesions (for diagnosis or palliation of symptoms), three patients had liver‐directed therapies (chemoembolization or Y90 radioembolization), six patients had radiation treatments (RT) to the primary tumors, one patient had RT to his liver metastases, five patients had RT to bone lesions, two patients had RT for peritoneal disease, and two patients received RT for brain metastases. Given the small number of patients that received local therapies, we were not able to evaluate trends in this population.

### 3.4 Overall survival

Of the 121 total patients included, 33% (*n* = 41) were alive at 12 months, and 25% (*n* = 31) were alive at 18 months. The Median OS of the entire cohort was 8.9 months. Factors that did not influence OS included: CCI, location of the primary disease, presence of lymph nodes, number of metastatic lesions (≤3, 4–6, 6–10, >10), age, or gender. There was no difference in OS among patients who received first‐line therapy with FOLFIRINOX versus gemcitabine‐based therapies.

**FIGURE 1:**
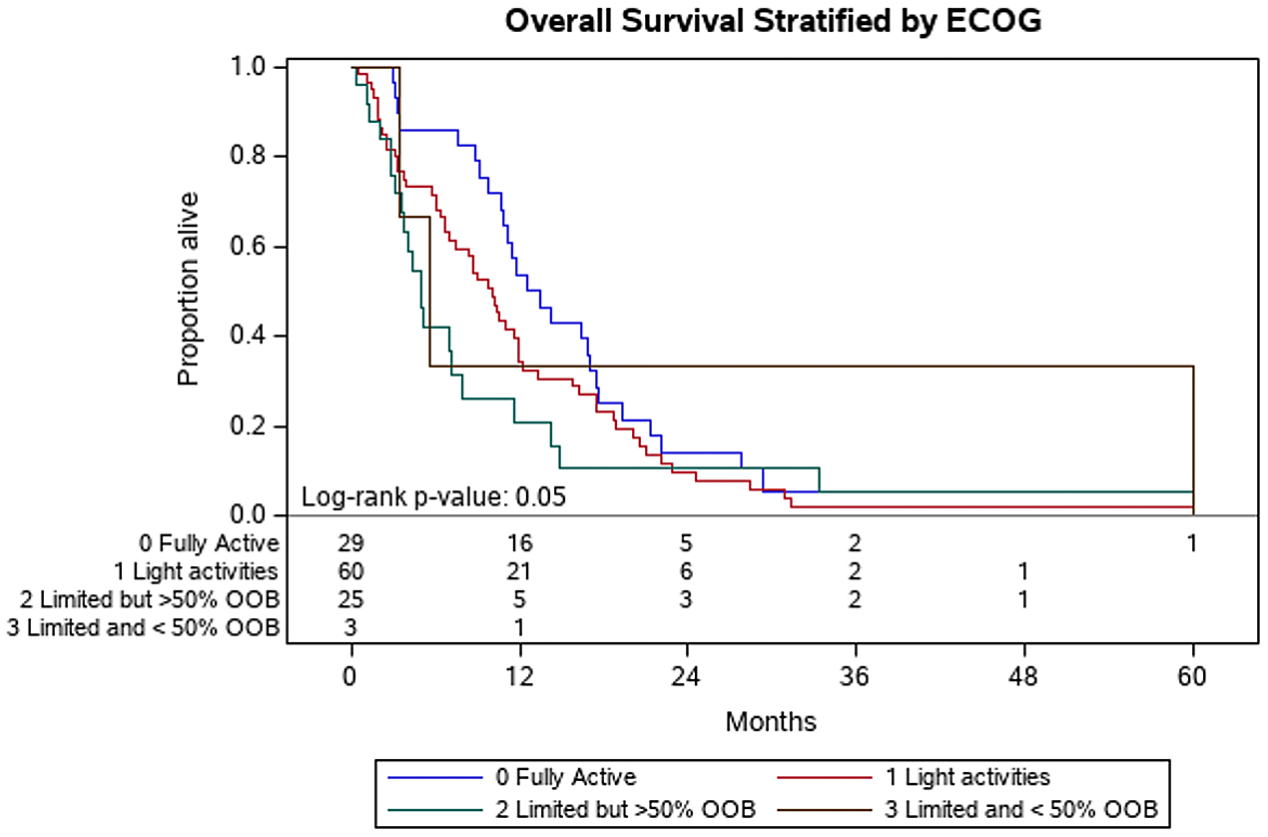
Kaplan–Meier plot of patients stratified by ECOG status: 0‐fully active, 1‐light activities, 2‐limited but spends >50% out of bed, 3‐limited and spends <50% of time out of bed. Probability of death at Year 1 was 54% for patients with ECOG 0 and 34% for patients with ECOG 1 (*p* = 0.05).

**FIGURE 2:**
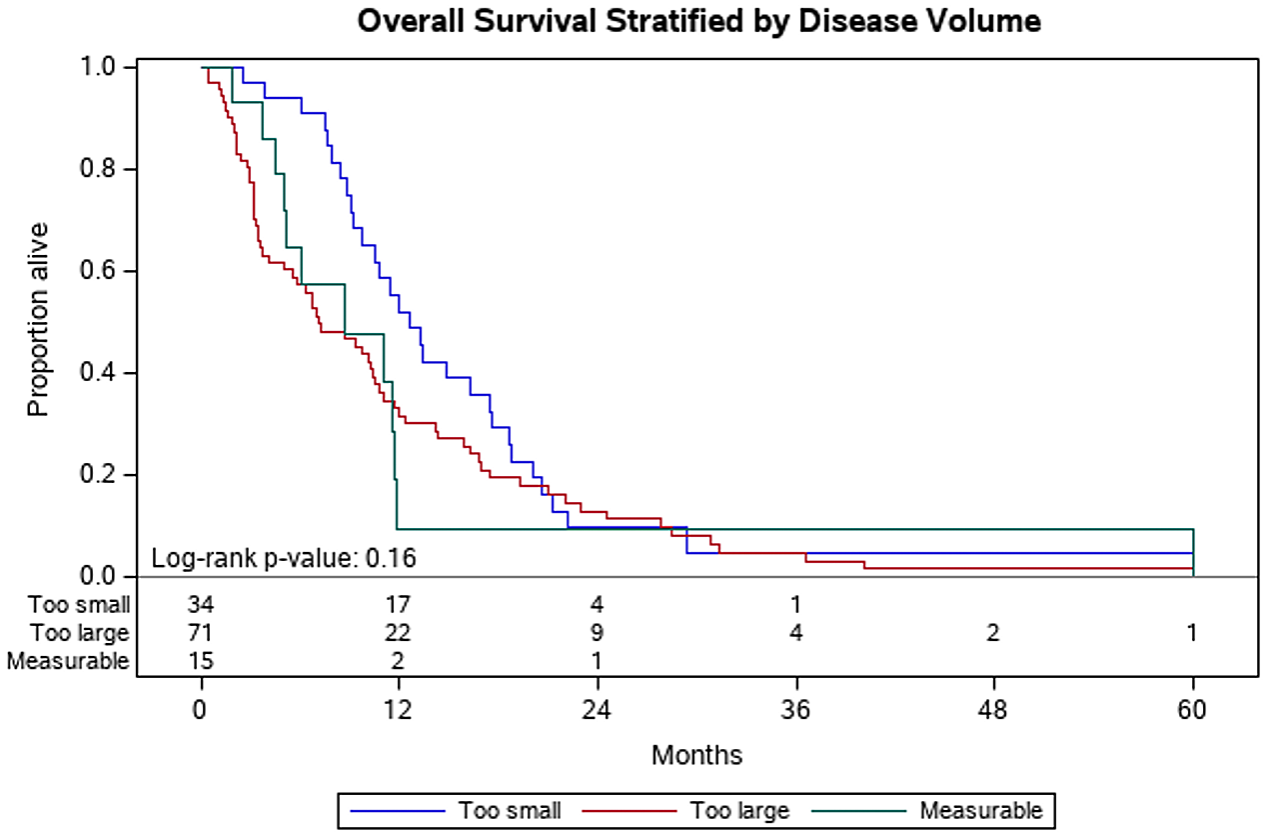
Kaplan–Meier plot for overall survival stratified by disease volume: too small (<0.2 cc), measurable, too large (>60.7 cc). Probability of death at Year 1 was 52% for patients with tumors <0.2 cc, compared to 32% for tumors >60 cc (*p* = 0.004).

**FIGURE 3:**
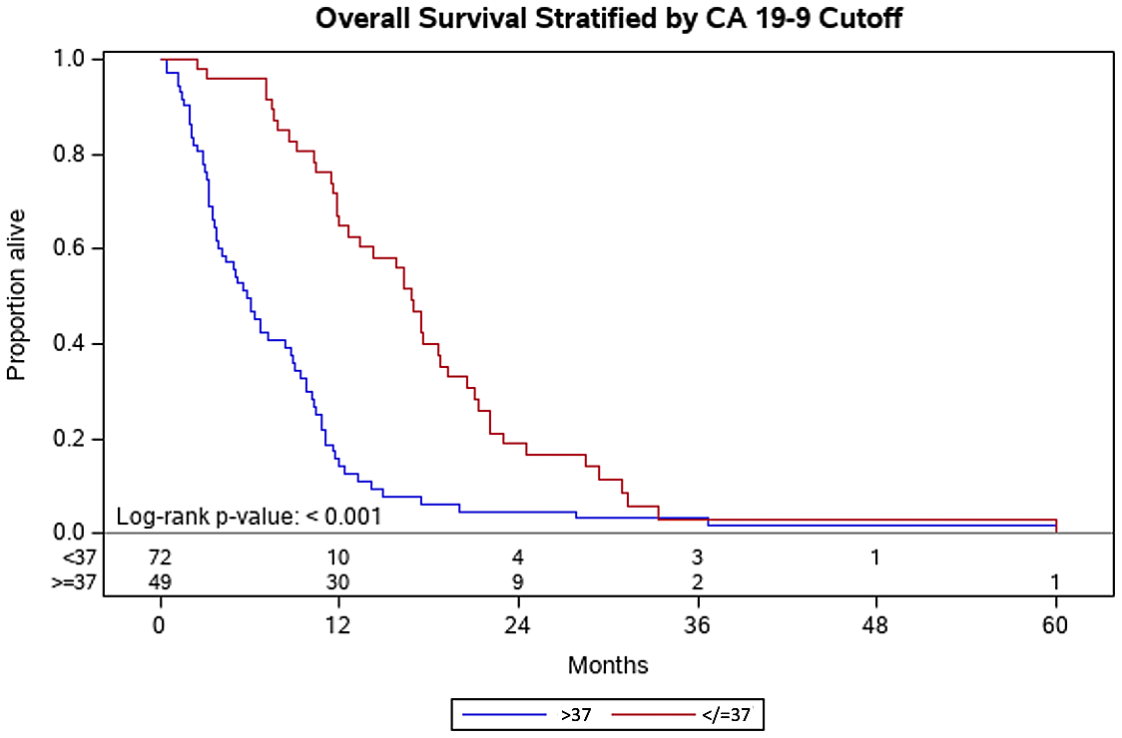
Kaplan–Meier plot for overall survival for patients that normalized their CA 19‐9 (<37) compared to those that did not. Probability of death at Year 1 for patients that normalized their CA 19‐9 was not evaluable due to small number of patients, compared to 65% for those that did not normalize their CA 19‐9 (*p* < 0.001). This difference remained significant at Year 2 (*p* = 0.03).

**FIGURE 4:**
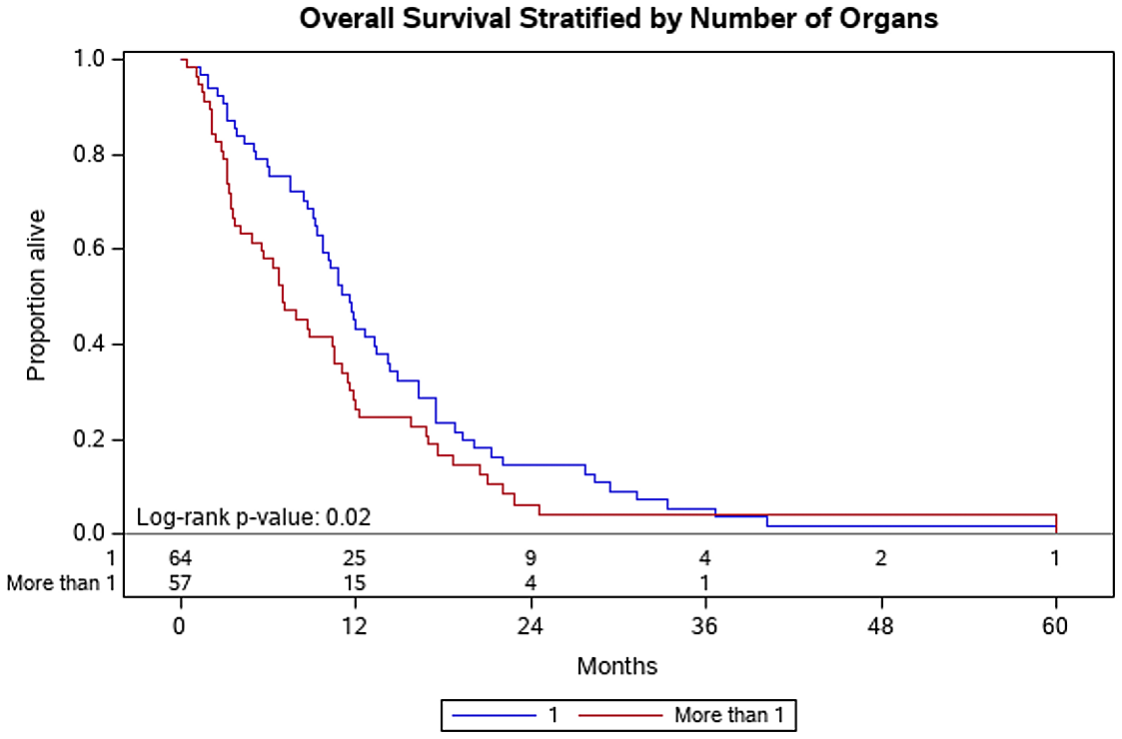
Kaplan–Meier plot for overall survival stratified by number of organs with metastasis (1 compared to >1). Probability of death at Year 1 was 43% for patients with only one organ with metastasis, compared to not evaluable for patients with more than one organ with metastases (*p* = 0.06).

**FIGURE 5:**
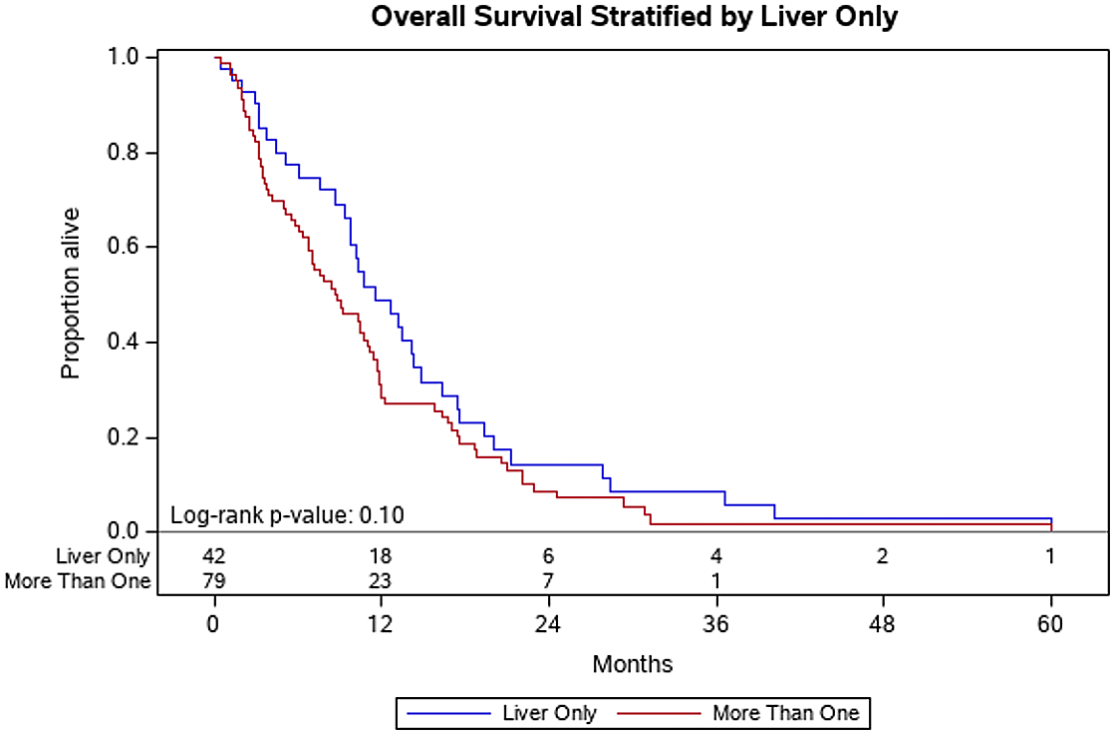
Kaplan–Meier plot for overall survival stratified by disease in the liver only or liver with additional sites. Probability of death at Year 1 was 49% for patients with only liver only metastasis, compared to 28% for patients with metastasis in the liver and additional sites (*p* = 0.04).

Factors associated with improved OS at Year 1 included: patients with ECOG of 0 at diagnosis (ECOG 0 vs. ECOG 1, *p* = 0.04) (Figure 1) and disease volume (<0.2 cc vs. > 60 cc, *p* = 0.004) (Figure 2). Normalization of the CA 19‐9 (<37) was significantly associated with improved OS at Year 1 (*p* < 0.001) (Figure 3). Having metastasis involving only one organ showed a nearly significant improvement toward improved OS at Year 1; however, this did not reach formal statistical significance (*p* = 0.06) (Figure 4). The presence of disease in the liver only was associated with improved OS at Year 1 (*p* = 0.04) (Figure 5). This difference was not seen in patients with lung only metastases or peritoneal implants.

At Year 2, the only predictor of OS for patients with elevated CA 19‐9 was normalization of the CA 19‐9 (*p* = 0.03) (Figure 3). In those patients that normalized their CA 19‐9, the median overall survival was 16 months. The total number of metastatic lesions, presence of lymph nodes and location of the pancreatic primary tumor were not significant predictors for OS.

### 3.5 Landmark analysis at 18 months

With the goal of understanding the differential characteristics of those patients with exceptional OS, a landmark analysis was performed at 18 months. This analysis compared patients with survivals less than 18 months to those with survivals greater than 18 months. The results of this analysis demonstrated a difference in the presence of lung only metastases (36% vs. 16%, *p* = 0.04), the number of organs with metastases (≥2 vs. 1, *p* = 0.04), and disease volume (mean of 19.1 cc vs. 1.4 cc, *p* = 0.04) between patients surviving <18 months and ≥18 months, respectively.

## 4 DISCUSSION

In this exploratory study, we comprehensively analyzed patients with histologically confirmed metastatic PDAC, who were treated with modern standard of care systemic therapy. Such chemotherapies included 5‐fluorouracil and gemcitabine‐based regimens. We selected a cohort of patients for whom detailed clinical outcomes were available, with long‐term follow‐up, along with diagnostic imaging that was accessible for individual patient level segmentation. Several factors were identified that were associated with improved OS outcomes. These factors included normalization of CA 19‐9, small tumor volume, and the number of organs with metastatic disease. Such criteria confirms clinical suspicions regarding patients with metastatic PDAC who may have improved outcomes. These may be helpful for the design of interventional trials focused on management strategies for patients with metastatic PDAC.

There is one other series that has recently evaluated the presence of an oligometastatic state in patients with PDAC. This example was from Damanakis et al., who proposed a definition of the oligometastatic state that includes the presence of ≤4 metastasis in liver or lung, CA 19‐9 baseline of <1000 U/mL, and response or stable disease after first‐line chemotherapy. This group of patients had a median survival of 19.4 months, compared to 7.2 months in the remaining patients.^6^ There are some important differences between our cohort and that presented by Damanakis et al. First, in their series nearly all patients were treated with gemcitabine‐based therapy and 63% of patients had single organ metastases. They also excluded patients who underwent local therapies. In our cohort, we found that approximately 40% of patients received first‐line treatment with FOLFIRINOX. Nonetheless, we did not identify any difference in OS when comparing the use of FOLFIRINOX or gemcitabine‐based chemotherapies. This is consistent with recently published prospective trials evaluating the use of chemotherapy in the metastatic setting.^20^

In our cohort, we have identified novel characteristics associated with improved OS. We saw that patients with normalized CA‐19‐9 had a median OS of 16 months, regardless of the initial volume of disease. We also found that a limited volume of metastatic disease (<0.2 cc) was associated with improved OS. Limited tumor volume and having involvement of only one organ, was a more powerful predictor of OS than having four or fewer metastatic lesions, or CA 19‐9 less than 1000 U/mL. Notable is that 25% of the patients collected in our series were alive at 18 months. Interestingly, these patients approach the median OS seen in patients with the early stages of pancreatic cancer.^21^ We hypothesize that the biology of this unique cohort can be explained by a combination of limited volume of disease combined with response to therapy.^6^ Biological response, as reflected by CA 19‐9, is known to be one of the most important factors to predict outcomes in PDAC.^22^, ^23^ Also, CA 19‐9 may indicate response to chemotherapy in patients with metastatic PDAC before the response is evident in radiological imaging.^24^ By evaluating treatment characteristics, we identified that normalization of CA‐19‐9 remains one of the most important factors for OS at Years 1 and 2. This is consistent with other reports, such as by Damanakis et al., who found that having a CA 19‐9 <1000 U/mL was associated with a significant improvement in OS (7.7 months vs. 16 months).^6^

The identification of characteristics associated with improved clinical outcomes raises the question as to the potential role of more aggressive interventions in selected patients. Specifically, should patients with metastatic disease ever be given consideration for more aggressive local interventions, or metastatic‐directed interventions, such as radiation therapy or surgical resection? Given the small number of patients, the presence of local interventions in our cohort was not seen to reflect an improvement in OS. This conflicts with other recent publications that have introduced this hypothesis.^25^ However, our group of patients who underwent local interventions was very small, and therefore the findings in this cohort are not reliably interpretable.

The question remains if PDAC is often considered a systemic disease at the time of diagnosis, should the presence of radiological evidence of metastatic disease be such a distinguishing feature in global management? Is there a role for local interventions in the setting of a clinical trial with metastatic disease? This may represent a novel and important area of research that should be considered for patients with metastatic PDAC. Along these lines, it has been shown in other solid tumors, such as non‐small cell lung cancer, that OS can be improved with local treatments of the primary cancer or treatment to sites of metastatic disease.^15^, ^16^, ^17^ Important features of these studies are that they were prospective and randomized, which is an absolutely critical detail to avoid retrospective selection bias.

Retrospective data have suggested that resection of the primary tumor and solitary liver metastases can potentially improve overall survival in PDAC.^18^, ^19^ While we are not ready to endorse this approach, excellent local control can be obtained with SBRT in oligometastatic pancreatic cancer, with an impressive median OS of 23 months.^26^ These results were also seen in a retrospective cohort from China.^25^ These encouraging results suggest that PDAC may harbor an oligometastatic state in a very carefully selected subset of patients. However, the specific criteria for patient selection for such trial design remains poorly understood.

This study has limitations intrinsic to a retrospective review. Such limitations include missing data, selection bias, and a relatively small cohort size. Our goal was to limit this data collection to only those patients with detailed imaging data available for segmentation, along with adequate follow‐up to assess for patient outcomes. This results in a relatively small series, and data recompilation was limited by information collected by the clinicians at the time of diagnosis. Prospective, randomized research is needed to properly study this group of patients that might benefit from more aggressive local interventions.

Recently published and ongoing trials clearly hint at a benefit for neoadjuvant therapy in non‐metastasized PDAC.^27^, ^28^, ^29^ Even though these studies do not include patients with metastatic disease, we could consider a similar approach for patients with metastatic disease, where patients with good response to systemic therapy and normalization of CA‐19‐9 are selected for consideration of additional local therapies. In our cohort, we observed that patients who lived >18 months were likely to have received more cycles of chemotherapy and experienced normalization of CA 19‐9. Evaluating the ability to tolerate systemic therapies and having a good response to these agents can help identify those patients that might benefit from additional treatments and spare excess toxicities to patients that will not receive such benefit.

## AUTHOR CONTRIBUTIONS

**Saryleine Ortiz de Choudens:** Conceptualization (lead); data curation (lead); formal analysis (lead); investigation (lead); methodology (lead); project administration (lead); writing – original draft (lead); writing – review and editing (lead). **Alexis Visotcky:** Formal analysis (equal); writing – review and editing (supporting). **Anjishnu Banerjee:** Conceptualization (equal); formal analysis (equal); writing – review and editing (supporting). **Mohammed Aldakkak:** Data curation (equal); project administration (equal); resources (equal); software (equal); writing – review and editing (supporting). **Susan Tsai:** Conceptualization (equal); data curation (equal); writing – review and editing (supporting). **Douglas B. Evans:** Conceptualization (equal); data curation (equal); writing – review and editing (supporting). **Kathleen K. Christians:** Conceptualization (equal); data curation (equal); writing – review and editing (supporting). **Callisia Clarke:** Conceptualization (equal); data curation (equal); writing – review and editing (supporting). **Ben George:** Conceptualization (equal); data curation (equal); writing – review and editing (equal). **Aditya Shreenivas:** Conceptualization (equal); data curation (equal); writing – review and editing (supporting). **Mandana Kamgar:** Conceptualization (equal); data curation (equal); writing – review and editing (supporting). **Sakti Chakrabarti:** Conceptualization (equal); data curation (equal); writing – review and editing (equal). **Kulwinder Dua:** Conceptualization (equal); data curation (equal); writing – review and editing (equal). **Abdul Khan:** Conceptualization (equal); data curation (equal); writing – review and editing (supporting). **Srivats Madhavan:** Conceptualization (equal); data curation (equal); writing – review and editing (supporting). **Beth Erickson‐Wittmann:** Conceptualization (equal); data curation (equal); resources (equal); supervision (equal); writing – review and editing (equal). **William A. Hall:** Conceptualization (equal); data curation (equal); formal analysis; investigation (equal); methodology (equal); project administration (equal); resources (equal); supervision (equal); validation (equal); visualization (equal); writing – original draft (equal); writing – review and editing (equal).

## FUNDING INFORMATION

This research received no specific grant from any funding agency in the public, commercial, or not‐for‐profit sectors.

## CONFLICT OF INTEREST STATEMENT

William A Hall is a consultant for Atkis Oncology and receives travel/research funding from Elekta. Aditya Shreenevas is a consultant for Taiho Oncology, and receives research support from Natera and CARIS. MandanaKamgar receives research funding from Cornerstone Pharmaceuticals. Ben George is a consultant for Ipsen, Bristol Myers Squibb, Foundation Medicine, Taiho Oncology, Eisai, BTG (Boston Scientific), Roche/Genentech, and Pfizer. He receives research support from Foundation Medicine (Inst), Roche/Genentech (Inst), Hoffman La‐Roche (Inst), Taiho Oncology (Inst), Boehringer Ingelheim (Inst), Toray, Hutchison Medipharma, Mirati Therapeutics, Sirnamomics (Inst), Trishula Therapeutics (Inst), Transcenta (Inst), Carsgen (Inst), Pfizer (Inst), Helix Biopharma (Inst), Glyconex (Inst), Faeth Therapeutics (Inst) and has stock ownership of XBiotech. The remainder of the authors have no conflicts of interest to disclose.

## ETHICS STATEMENT

This project was approved by the Medical College of Wisconsin institutional review board (PRO00037616).

## Data Availability

The data that support the findings of this study are available from the corresponding author, upon reasonable request
